# Combinatorial supplementation of fish feeds enhanced growth performance and disease resilience in aquaculture

**DOI:** 10.1186/s40104-026-01357-3

**Published:** 2026-03-08

**Authors:** The-Thien Tran, Manish Mahotra, Kaarunya Sampathkumar, Wenrui Li, Hong Yu, Ling Xin Yong, Li Ling Tan, Mingyue Sun, Patricia Lynne Conway, Say Chye Joachim Loo

**Affiliations:** 1 School of Materials Science and Engineering, Nanyang Technological University, 50 Nanyang Avenue, Singapore, 639798 Singapore; 2 Singapore Centre for Environmental Life Sciences Engineering (SCELSE), Nanyang Technological University, 60 Nanyang Drive, Singapore, 637551 Singapore; 3 School of Biological, Earth and Environmental Sciences, The University of New South Wales, Sydney, NSW 2052 Australia; 4 Lee Kong Chian School of Medicine, Nanyang Technological University, 59 Nanyang Drive, Singapore, 636921 Singapore

**Keywords:** Asian seabass fingerlings, Curcumin, Encapsulation, Pathogens, Probiotics, Productivity

## Abstract

**Background:**

Aquaculture has grown rapidly in recent decades, yet recurrent bacterial disease outbreaks continue to cause severe economic losses and fuel concerns over antibiotic resistance. With antibiotic use increasingly restricted, sustainable disease management strategies are urgently required. Probiotics and natural bioactive compounds, such as curcumin, have emerged as promising alternatives, but their combined application remains underexplored.

**Results:**

We evaluated the co-administration of encapsulated probiotics and curcumin in functional feeds on growth performance and disease resilience of Asian seabass (*Lates calcarifer*) fingerlings challenged with *Streptococcus iniae* and *Vibrio parahaemolyticus*. Among 11 probiotic strains screened, *Lactiplantibacillus plantarum* displayed the strongest inhibitory effect and highest viability following alginate-based encapsulation. Curcumin selectively inhibited pathogens without affecting probiotic growth, and synergistic antimicrobial effects were observed when combined with probiotics. Feeding trials showed that encapsulated probiotics increased body weight by 33% compared with controls. Diets supplemented with probiotics, curcumin, or their combination significantly improved feed conversion efficiency and survival. Notably, co-supplementation yielded the greatest benefits, achieving the highest survival rates under pathogen challenge and enhancing immune protection beyond individual treatments.

**Conclusions:**

These findings demonstrate that probiotics combined with curcumin constitute a natural, antibiotic-free strategy to improve fish growth and disease resistance. This functional feed approach provides a scalable and sustainable platform for advancing responsible aquaculture and may inform broader applications in animal production systems.

**Supplementary Information:**

The online version contains supplementary material available at 10.1186/s40104-026-01357-3.

## Introduction

Global fish production from aquaculture reached approximately 126 million tons in 2023, with an estimated value of USD 296.5 billion [1]. Over the past few decades, while capture fisheries have remained relatively stable, aquaculture has experienced substantial growth. Between 1990 and 2018, global aquaculture production increased by 527%, accompanied by a 122% rise in total fish consumption during the same period [2]. However, despite these advancements, an estimated 35% of total fisheries and aquaculture production is lost or wasted annually. A significant contributor to these losses is the widespread occurrence of bacterial infections and disease outbreaks, which place considerable economic pressure on the industry [3]. The global economic loss due to aquaculture-related diseases is estimated to exceed USD 6 billion annually [4], with common pathogens such as *Streptococcus*, *Vibrio*, and *Aeromonas*, accounting for approximately USD 1 billion of this total [5].

For the past five decades, antibiotics have served as the primary method for controlling infectious diseases in aquaculture [6]. However, this practice has contributed to the emergence of antibiotic-resistant bacteria and the spread of transferable resistance genes in both fish pathogens and commensal bacteria within aquatic environments. These resistance genes can be disseminated through horizontal gene transfer, potentially reaching human pathogens and posing significant public health risks [7–9]. As a result, the use of antibiotics in aquaculture has been banned or heavily restricted in many regions, including the European Union, the United States, and China [10, 11]. These concerns have prompted increased interest in developing sustainable and environmentally friendly alternatives for disease control in aquaculture systems.

One promising strategy involves the incorporation of probiotics into feed for aquaculture. Probiotics are defined as “live microorganisms which, when administered in adequate amounts, confer a health benefit on the host” [12]. Since their initial application in aquaculture in 1986, probiotics have been demonstrated to improve growth performance, enhance immune responses, and increase resistance to infectious diseases [13–17]. Commonly used probiotics in aquaculture include members of the phylum Firmicutes, particularly lactic acid bacteria (LAB) such as *Lactococcus*, *Lactobacillus*, and *Bacillus* species, which are applied either as single strains or in combination. These probiotics exert their beneficial effects through various mechanisms, including gut colonization, production of antimicrobial compounds, competition with pathogens for nutrients and adhesion sites, and the enhancement of nutrient absorption and host metabolism [18–21].

Specific LAB strains such as *Lactococcus lactis* CLFP 101, *Lactiplantibacillus plantarum* CLFP 238, and *Limosilactobacillus fermentum* CLFP 242 have demonstrated inhibitory activity against pathogens including *Aeromonas hydrophila*, *Aeromonas salmonicida*, *Yersinia ruckeri*, and *Vibrio anguillarum*. Moreover, these probiotics contribute to the production of digestive enzymes and bioactive compounds that promote fish growth and health [22–26].

In addition to probiotics, the use of natural bioactive compounds, often referred to as nutraceuticals, has gained traction in the development of sustainable aquaculture practices [27, 28]. Among these, curcumin—a polyphenolic compound derived from turmeric (*Curcuma longa*), has received significant attention for its diverse biological activities, including antioxidant, anti-inflammatory, antimicrobial, immunomodulatory and antiparasitic effects [29, 30]. Curcumin supplementation has been shown to improve growth performance, hematological parameters, immune function, antioxidant capacity, and disease resistance in aquaculture species [31–34]. It has demonstrated antibacterial activity against common pathogens such as *A. hydrophila* and *Aeromonas sobria* [35], and has been reported to enhance survival and disease resistance in shrimp infected with *Vibrio harveyi* [36], silver catfish exposed to *Streptococcus agalactiae* [33], and rainbow trout challenged with *A. salmonicida* subsp. *achromogenes* [31].

Despite the demonstrated benefits of both probiotics and curcumin, most studies have examined their effects independently. There is a notable lack of research exploring their combined application, which could potentially yield synergistic effects. It was thereby hypothesized that co-administration of probiotics and curcumin may enhance antimicrobial activity, reduce the required effective dose of curcumin, or improve probiotic performance—resulting in more effective and cost-efficient disease control in aquaculture. Accordingly, the objective of this study was to evaluate the synergistic effects of co-supplementing encapsulated probiotics with curcumin on fish health, immune and antimicrobial responses, and disease resistance, in order to develop a more sustainable alternative to antibiotics in aquaculture.

To enhance the effectiveness of this combinatorial approach, our study investigated the encapsulation of probiotics for co-administration with curcumin. Encapsulation technology offers several advantages for probiotic delivery, including protection during storage, improved survival through the gastrointestinal tract, and targeted delivery to the gut for enhanced colonization [37]. Moreover, the combination of encapsulated probiotics with curcumin may produce synergistic effects, thereby improving the overall functional performance of the formulation. This strategy aims to optimize the stability and bio-efficacy of the probiotic component while also harnessing the known health-promoting properties of curcumin. Ultimately, the co-supplementation of encapsulated probiotics and curcumin presents a promising approach for the development of functional aquaculture feeds that promote sustainable disease control and reduce reliance on antibiotics in aquaculture.

## Materials and methods

### Materials

Probiotic strains used in this study include 12 strains from 12 species, namely *Lactobacillus acidophilus* (LAS), *Lacticaseibacillus paracasei* (LPI), *Limosilactobacillus reuteri* (LRI), *Limosilactobacillus amylovorus* (LAM), *Limosilactobacillus oris* (LOS), *Lactiplantibacillus plantarum* (LPM), *Limosilactobacillus fermentum* (LFM), *Liquorilactobacillus satsumensis* (LSS), *Lactobacillus helveticus* (LHS), *Lentilactobacillus kefiri* (LKI), *Lentilactobacillus hilgardii* (LHI), and *Lacticaseibacillus rhamnosus* GG (LGG), as detailed in Additional file 1: Table S1. Of which, strains LAS, LRI, LAM, LOS, LPM, LFM and *Escherichia coli* K12 were obtained from PC Biome Pte. Ltd., Singapore. Strains LPI, LSS, LHS, LKI, LHI, were isolated from milk and water kefir, as reported in Tan et al. [38], and *L. rhamnosus* GG was isolated from a purchased Culturelle^®^ probiotic pill (i-Health, Inc., Crownwell, USA). *E. coli* Nissle 1917 was isolated from a purchased Mutaflor^®^ capsule (Amber Compounding Pharmacy, Singapore). Two aquaculture pathogens used in this study, including *Vibrio parahaemolyticus* ATCC 17802 and *Streptococcus iniae* ATCC 29178 were purchased from ATCC, USA.

De Man, Rogosa and Sharpe (MRS), Nutrient Broth (NB), Wilkins-Chalgren Anaerobe (WC) media were purchased from Thermo Fisher Scientific, USA. Bacto agar was purchased from BD, USA. All other chemicals used in this experiment, including curcumin from *Curcuma longa* and dimethyl sulfoxide (DMSO), were purchased from Sigma Aldrich, USA.

### Growth of microorganisms

Probiotics were inoculated in MRS media and incubated according to respective conditions, under aerobic or anaerobic environments, as indicated in Additional file 1: Table S1. For anaerobic conditions, strains were handled within the Bactron 300 anaerobic chamber (Sheldon Manufacturing, USA). All pathogens were inoculated in WC broth, with exception of *V. parahaemolyticus*, which was inoculated in NB supplemented with 3% (w/v) NaCl. Pathogens were incubated aerobically at 30 °C conditions for 24 h prior to use.

### Antimicrobial susceptibility testing (AST)

#### Screening probiotics against *S. iniae* and *V. parahaemolyticus*

The inhibitory potential of probiotic strains against aquaculture pathogens was evaluated using the agar well diffusion assay. Confluent pathogen cultures were diluted in WC medium based on their optical density at 600 nm (OD_600_). *V. parahaemolyticus* and *S. iniae* were adjusted to an OD_600_ of 0.5. A 100 µL aliquot of the diluted pathogen suspension was spread onto WC agar plates, and 6 mm-diameter wells were created using the back of a 200-µL pipette tip. Each well was filled with 50 µL of probiotic culture and the plates were incubated at 30 °C for 24 h. After incubation, inhibition zones, defined as clear areas devoid of pathogen growth, were recorded as evidence of probiotic-mediated inhibition. The inhibition zone size was measured as the distance from the edge of the well to the outer boundary of the clear zone.

#### Assessing the compatibility of probiotics and pathogens with curcumin

An agar dilution method was used to assess the compatibility of probiotics and pathogens with curcumin, ensuring that the selected probiotics were not inhibited by the presence of curcumin and evaluating whether curcumin exhibited inhibitory activity against *V. parahaemolyticus* and *S. iniae*. Briefly, probiotic and pathogen inocula were diluted to an OD_600_ of 0.1 and 0.5 in MRS and WC broths, respectively. A 100 µL aliquot of each diluted culture was spread onto MRS or WC agar plates. A range of curcumin concentrations (5 mg/mL to 0.01 mg/mL prepared in DMSO) was prepared by serial dilution and an aliquot (3 µL) drop dispensed onto bacterial lawns on the MRS and WC agar surfaces. After 24 h incubation at 30 °C, plates were examined for the presence of inhibition zones. Complete inhibition was defined as the absence of bacterial colonies beneath the curcumin or DMSO droplet, while partial inhibition was indicated by visible thinning of the bacterial lawn in the affected area. DMSO at corresponding concentrations ranging from 12.5% to 0.025% was also tested as control samples.

#### Evaluating synergistic effects of probiotics and curcumin on pathogens

The synergistic effects of probiotics and curcumin against pathogens were evaluated using the agar well diffusion method. Based on the results of the initial screening against *S. iniae *and* V. parahaemolyticus*, four probiotics — *L. paracasei* spp., *L. helveticus* sp., *L. rhamnosus* GG, and *L. plantarum* sp. — were selected for further testing. These strains were assessed in combination with curcumin at concentrations of 0.75 mg/mL and 1.5 mg/mL. These concentrations were determined based on prior compatibility testing, representing the lowest concentration effective against *V. parahaemolyticus* and *S. iniae*, respectively.

Pathogen cultures were prepared by diluting them to an OD_600_ of 0.5. Curcumin stock solution was then added to the pathogen cultures to achieve final curcumin concentrations of 1.5 mg/mL or 0.75 mg/mL. The pathogen-curcumin mixtures (100 µL) were spread onto WC agar plates. Wells were created in the agar, and 50 µL of the selected probiotic cultures were added to each well. The plates were incubated at 30 °C for 24 h. After incubation, the plates were examined for the presence of inhibition zones. The size of the inhibition zones was compared to results from the individual probiotic assays to assess whether combined supplementation of curcumin and probiotics enhanced the inhibitory effects on the pathogens.

### Encapsulation of probiotics

Four probiotic strains that showed synergistic effects with curcumin against the pathogens were then encapsulated using a patented technique [39]. Briefly, the probiotic cultures were grown in MRS broth at 30 °C for 24 h, then washed three times with 0.9% (w/v) NaCl solution to remove the residual media. The cells were then suspended in alginate solution containing protectants to prevent loss during spray drying. The alginate cell suspension was then spray dried together with a cross-linking agent. The encapsulated probiotics were characterized using SEM to observe the shape and size of the microparticles. The survivability of the probiotics after spray drying was assessed by dissolving the spray dried microparticles in 50 mmol/L sodium citrate, followed by drop-plating onto MRS agar. The probiotics were also subjected to simulated gastric fluid (SGF) (pH ~ 2) to mimic the environment in the gastrointestinal tract (GIT). The SGF was prepared with 0.2 mol/L NaCl and 2,000 units/mL porcine pepsin, with the pH adjusted to 2 using HCl [40]. All experiments were conducted in three biological replicates.

For the considered end application as supplements in fish feeds, the encapsulated probiotics must display good storage stability to enable the feeds to be stored at the aquaculture farms. The encapsulated probiotics were stored at 4 °C over a period of 12 weeks and the survivability was tested every four weeks to monitor the loss over the storage period. In addition, aliquots of samples during the storage period were also subject to acid exposure tests under SGF conditions to mimic and assess the viability of probiotics when taken up by fish. All storage stability and SGF exposure assays were performed in three biological replicates.

### Feeding trial with probiotic encapsulation and curcumin co-supplementation

#### Pelletizing fish feed for fingerlings

Experimental diets were formulated with defined inclusion levels of probiotics and curcumin based on previously reported effective ranges in fish. Probiotics were included at 1% (w/w) as an encapsulated powder, providing approximately 6.7 × 10^9^ CFU/kg diet, a dosage widely reported to enhance growth performance, gut health, and immune responses in Asian seabass and other teleost species [41–44]. Curcumin has been used across a wide range of dosages in fish trials (0.005%–4%, w/w), and the 0.5% (w/w) dose used in this study represents a biologically effective mid-range level that has been reported to enhance antioxidant capacity, digestive enzyme activity, nutrient utilization, and growth performance in various fish species without adverse effects [31, 45–48].

A fish feed pelletizer was used to produce 1.5–2 mm feed pellets suitable for fingerlings in a scalable and cost-effective manner. The basal diet was formulated with essential nutrients and supplemented with encapsulated probiotics (1%, w/w) and curcumin (0.5%, w/w), while a non-supplemented basal diet served as the control.

Prior to feed preparation, a preliminary assessment was conducted to evaluate the stability of non-encapsulated (free) probiotics (FP) in SGF. The free probiotic cells exhibited complete loss of viability after SGF exposure, indicating that they were unable to tolerate gastric conditions, whereas the encapsulated probiotics (EP) maintained good viability in SGF. Accordingly, free probiotics would not remain viable before reaching the intestine and were therefore not included as a standalone dietary treatment in the feeding trial. Instead, it was incorporated only into the FPFC diet, in which free probiotics were combined with free curcumin, to allow comparative evaluation with the encapsulated combinations.

During the pelletization process, the EP diets were prepared by incorporating 1% (w/w) spray-dried EP powder into the feed formulation. The EP powder was first mixed thoroughly with the basal feed ingredients, and subsequently extruded under controlled conditions to ensure that the nutritional profiles of all experimental feeds were comparable (Additional file 1: Table S2). By adjusting the pelletizer settings, different pellet sizes could be produced to accommodate various developmental stages of fish. Standard fish feed was modified to include encapsulated probiotics and curcumin, while a non-supplemented feed served as the control to evaluate their effects on fish growth performance and resistance to pathogen challenges. All diets were subjected to compositional analysis by Eurofins Food Testing Singapore Pte. Ltd., a certified analytical laboratory. To ensure probiotic viability, fresh batches of EP-supplemented diets were prepared every two weeks, stored at 4 °C, and aliquoted daily during the trial.

#### Growth performance

The growth performance trial was conducted at the fish facility at Pontus Research in Singapore. Asian seabass fingerlings were fed with control diet for 2-week acclimation period in 500-L recirculation aquaculture systems (RAS) tanks. Thereafter, similar-sized fish were randomly distributed into 5 experimental groups (EP, FC, FPFC, EPFC and control) included in Table 1. A total of 1,500 fish were used to test five different diets. Each diet was assigned to three replicate tanks, with 100 fish per tank. The initial average weight of the fish was approximately 6 g. The fish were hand-fed three times daily to apparent satiation for 28 weeks. The water temperature was maintained at around 29 ± 1 °C throughout the trial. All procedures adhered to ethical guidelines approved by the Institutional Animal Care and Use Committee (IACUC Approval No.: SFA-MAC-2024-01). At the end of the feeding trial, fish were starved for 24 h for complete food digestion for accurate weight measurements. Weight gain (WG), specific growth rate (SGR), feed conversion ratio (FCR) and survival rates were evaluated, with the equations from Amer et al. [49]:

$$WG=\frac{FBW-IBW}{IBW} \times 100\%$$

$$SGR \left(\frac{BW}{day}\right)= \frac{lnFBW-lnIBW}{T} \times 100\%$$

$$FCR=\frac{FI}{WG}$$

where FBW = final body weight (g), IBW = initial body weight (g), T = duration of the trial in days, WG = wet weight gain (g) and FI = estimated feed intake (g).

**Table 1:** Different study groups for the feeding trial

Group No.	Name
1	EP
2	FC
3	FPFC
4	EPFC
5	Control

*EP* Encapsulated probiotics, *FC* Free curcumin, *FPFC* Free probiotics and free curcumin, *EPFC* Encapsulated probiotics and free curcumin. Control refers to the basal diet without functional additives

### Pathogen challenge test

#### Challenge test against *V. parahaemolyticus*

Following a 28-week feeding period with functional feeds, a subset of fish (*n* = 30) from each experimental group was transferred to the Animal Biosafety Level 2 (ABSL-2) facility for a disease challenge experiment. Fish from the control diet group were used to determine the 50% lethal dose (LD_50_) of the *V. parahaemolyticus* challenge via intraperitoneal (i.p.) routes. Each test group underwent challenge trials in triplicate using the predetermined LD_50_ dose and was monitored daily for up to 14 d [50]. The experiment was concluded earlier if any experimental group exhibited 50%–100% mortality. The control diet group, exposed to varying *V. parahaemolyticus* doses, exhibited an LD_50_ of approximately 10^8^ CFU/mL via the i.p. route. All procedures adhered to ethical guidelines approved by the Institutional Animal Care and Use Committee (IACUC Approval No.: 202303–186).

#### Challenge test against *S. iniae*

At the end of the feeding trial, the fingerlings were subjected to a challenge test against *S. iniae*, conducted in a manner similar to the *Vibrio* challenge. Briefly, fish (*n* = 18) from each test group were injected intraperitoneally with 0.1 mL of normal saline solution (NSS) containing the LD_50_ dose of *S. iniae* in triplicate. The LD_50_ was determined using moderate (~ 3.1 × 10^6^ CFU/fish) and high (~ 1.5 × 10^8^ CFU/fish) bacterial doses. The highest i.p. dose of *S. iniae* resulted in ~ 42% cumulative mortality. To facilitate infection, fish were sedated, and a 1-cm^2^ area of skin was scraped using a scalpel blade before i.p. injection of ~ 2.4 × 10^7^ CFU/fish. Mortality was recorded daily for 12 d, and the experiment was concluded earlier if any experimental group exhibited 50%–100% mortality. This study followed ethical protocols approved by the Animal Ethics Committee (IACUC) of Temasek Polytechnic (Approval No.: 202312-191A).

### Statistical analysis

All experiments were conducted in triplicate unless otherwise specified, and results are presented as mean ± standard deviation (SD). All statistical analyses were conducted using GraphPad Prism version 9.5 software. Differences among groups were assessed using one-way ANOVA followed by Tukey’s HSD post hoc test for multiple comparisons. Survival curves from pathogen challenge trials were compared using the Log-rank (Mantel–Cox) test. A *P* < 0.05 was considered statistically significant. Significance levels were denoted as follows: ^*^P < 0.05, ^**^P < 0.01, ^***^P < 0.001, ^****^P < 0.0001, ns—no significant difference.

## Results

### Selection of probiotics against *S. iniae* and *V. parahaemolyticus*

**Fig. 1:**
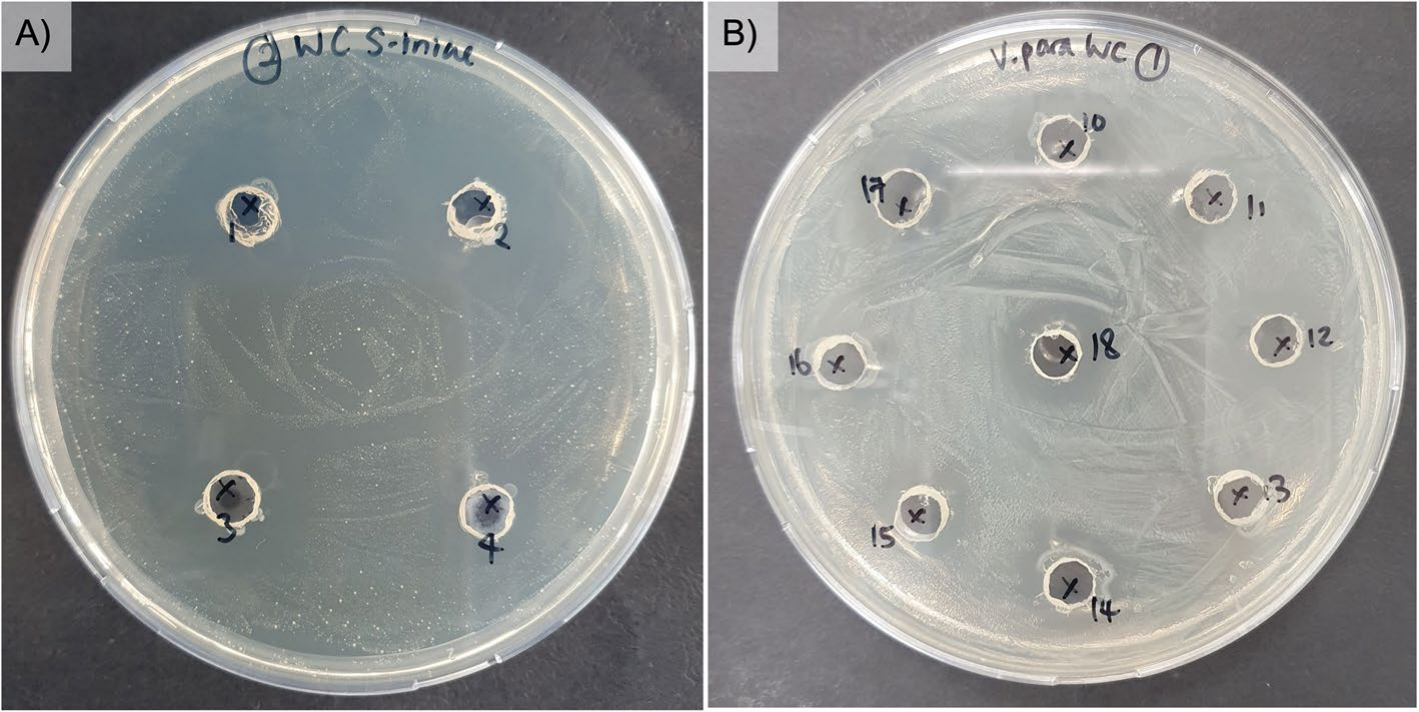
Agar well diffusion assay showing inhibition zones of selected probiotics against (**A**) *S. iniae* and (**B**) *V. parahaemolyticus*. The numbers labelled 1 to 18 in each well represent specific probiotic suspensions

A library of 11 probiotic strains and three control strains was screened for antagonistic activity against *S. iniae* and *V. parahaemolyticus*, as summarized in Table 2. Using the agar well diffusion assay (Fig. 1), 10 probiotic strains exhibiting strong antipathogenic activity were selected. In this assay, inhibition zones were classified as strong (+ + +) for diameters exceeding 4 mm, intermediate (+ +) for 2–4 mm, and weak (+) for those less than 2 mm.

**Table 2:** List of probiotics screened against *S. iniae *and *V. parahaemolyticus*

No.	Probiotics	Growth inhibition of *S. iniae*	Growth inhibition of *V. parahaemolyticus*
1	*Lactobacillus acidophilus* sp.	+ + +	+
2	*Lactobacillus paracasei* spp.	+ + +	+ +
3	*Lactobacillus reuteri* sp.	+ +	+ +
4	*Lactobacillus amylovorus* sp.	+ + +	+ +
5	*Lactobacillus oris* sp.	+ +	+
6	*Lactobacillus plantarum* sp.	+ + +	+ +
7	*Lactobacillus fermentum* spp.	+ +	+ +
8	*Lactobacillus satsumensis* spp.	+ + +	+ +
9	*Lactobacillus kefiri* sp.	+ + +	+ +
10	*Lactobacillus helveticus* sp.	+ + +	+ +
11	*Lactobacillus hilgardii* sp.	-	+ +
12	*L. rhamnosus* GG (positive control)	+ + +	+ +
13	*E. coli* Nissle 1917 (positive control)	-	-
14	*E. coli* K12 (negative non-probiotic control)	-	-

Each assay was performed in duplicate. Zones of inhibition were classified as strong (+ + +) when exceeding 4 mm, intermediate (+ +) when measuring 2–4 mm, weak (+) when less than 2 mm, and absent (-) when no detectable inhibition zone was observed [38]. Strain names labelled as sp. indicate a single species, whereas spp. indicates multiple species within the same genus [51]

To investigate the potential role of pH in the observed inhibitory effects, the pH of probiotic cultures was measured, ranging from 3.63 to 4.24. Additionally, MRS broth was adjusted to pH values between 3.0 and 6.2 for comparison. The results indicated that pH alone was not the primary determinant of probiotic antagonism. Notably, *S. iniae* was not inhibited in MRS broth at pH 3.5, despite this being more acidic than all tested probiotic cultures. Similarly, *V. parahaemolyticus* showed only weak inhibition at pH ≤ 5.0 in MRS broth, whereas most probiotic cultures demonstrated intermediate inhibition. The results suggest that the inhibitory activity of the probiotics was likely mediated by bioactive compounds rather than pH alone.

### Effects of curcumin on probiotics and pathogens

**Fig. 2:**
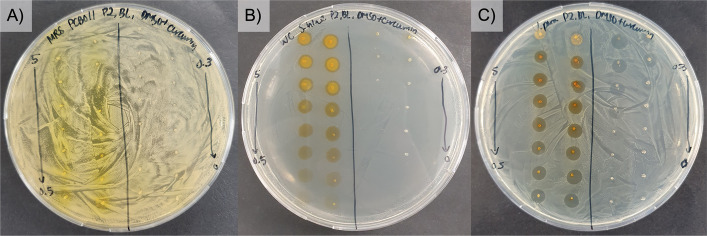
Bacteria lawn assay showing the effects of curcumin at different concentrations on probiotic strain (**A**) *L. plantarum* sp., (**B**) *S. iniae*, and (**C**) *V. parahaemolyticus*. The numbers on the agar plates indicate the tested curcumin concentrations (0–5 mg/mL; dissolved in DMSO)

To assess the compatibility of curcumin with probiotics, its potential inhibitory effects on probiotic growth were examined. Additionally, its antimicrobial activity against *S. iniae* and *V. parahaemolyticus* was evaluated. As shown in Fig. 2A, curcumin did not suppress the growth of the selected probiotic strains, indicating its suitability for co-supplementation.

In contrast, curcumin exhibited dose-dependent inhibitory effects against *S. iniae* and *V. parahaemolyticus*. For *S. iniae*, a complete growth inhibition was observed at concentrations of ≥ 1.5 mg/mL curcumin on agar, whereas inhibition occurred at concentrations ≥ 0.75 mg/mL curcumin for *V. parahaemolyticus* (Fig. 2B and C**)**. Accordingly, 0.75 mg/mL and 1.5 mg/mL were selected for subsequent experiments to evaluate potential synergistic effects, as these concentrations are consistent with the reported effective ranges for curcumin’s antimicrobial activity against pathogens [52].

### Combinatorial effects of curcumin and probiotics

The potential synergistic antimicrobial effects of curcumin in combination with probiotics were investigated using the agar well diffusion assay. Four probiotic strains—*L. paracasei* spp., *L. plantarum* sp., *L. helveticus* sp., and *L. rhamnosus* GG—were selected for co-administration with curcumin based on their demonstrated inhibitory effects against pathogens and compatibility with curcumin.

**Fig. 3:**
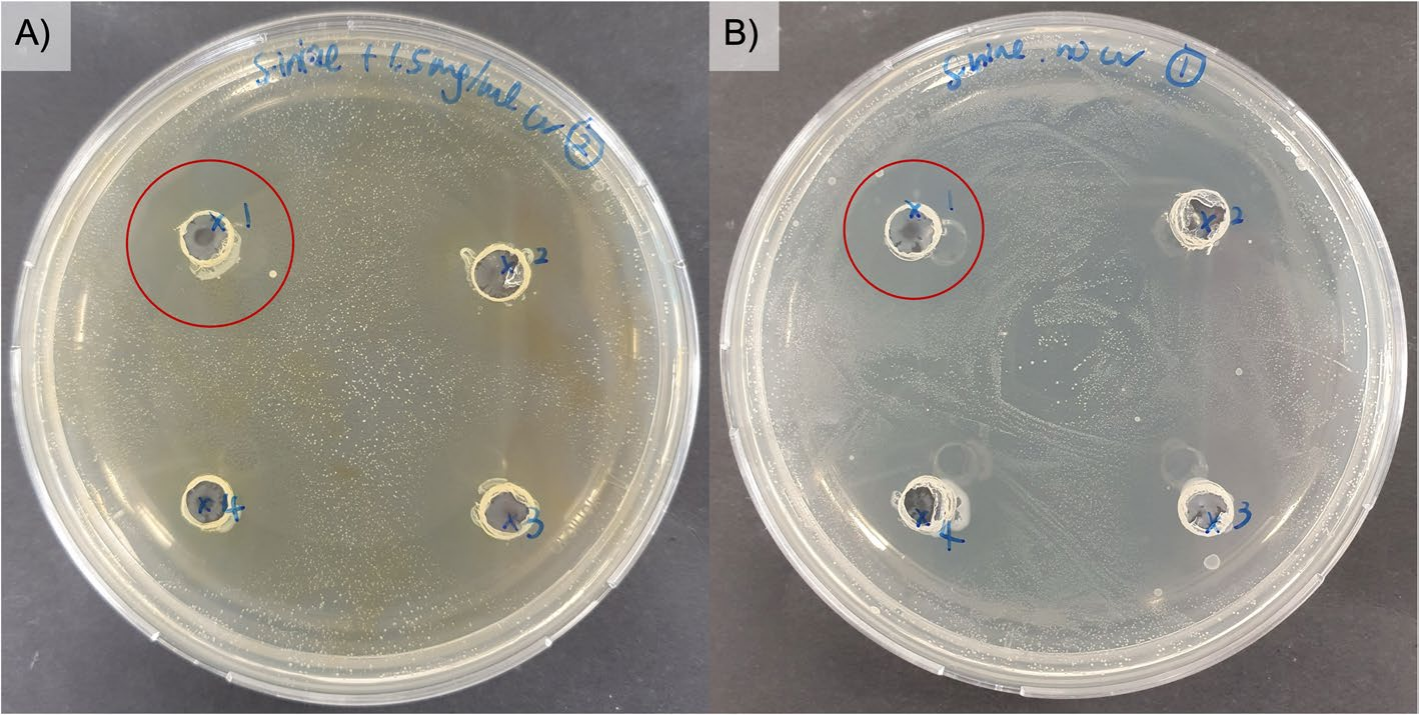
The inhibition zones in a *S. iniae* lawn of probiotics (**A**) with curcumin and (**B**) without curcumin. The numbers labelled 1 to 4 in each well represent specific probiotic suspensions

As illustrated in Fig. 3 and summarized in Table 3, the co-supplementation of curcumin with probiotics resulted in a clear synergistic inhibition of the pathogens. The combination generally produced greater antimicrobial effects than either curcumin or probiotics alone, as evidenced by larger inhibition zones. Specifically, a dose-dependent bactericidal effect was observed against *S. iniae*, with curcumin at 1.5 mg/mL producing a significantly larger zone of inhibition compared to 0.75 mg/mL. In the case of *V. parahaemolyticus*, the combination treatment resulted in noticeable thinning of the bacterial lawn across the plate, making it challenging to define and measure distinct inhibition zones.

**Table 3:** Combinatorial effects of curcumin and the four selected probiotics against *S. iniae*

		Size of inhibition zone, mm		
No.	Probiotics	Curcumin (mg/mL) + *S. iniae*		
		0 mg/mL	0.75 mg/mL	1.5 mg/mL
1	*L. paracasei* spp.	6.5	6.5	7.5
2	*L. plantarum* sp.	6	6.5	7
3	*L. helveticus* sp.	6	6	7.5
4	*L. rhamnosus* LGG	6	6.5	7

Values represent inhibition zone diameters (mm) measured in the presence of 0, 0.75, and 1.5 mg/mL curcumin. Results were calculated as an average of duplicate experiments. An increase in the size of the inhibition zone indicates a concentration-dependent effect

### Encapsulation of probiotics

To enhance the stability, survivability, and functional performance of sensitive probiotic strains, and to ensure their targeted delivery to the GIT, four probiotic strains—*L. paracasei* spp., *L. plantarum* sp., *L. helveticus* sp., and *L. rhamnosus* GG—exhibiting synergistic antimicrobial activity were selected for encapsulation. A patented spray-drying method was used to incorporate each strain into an alginate-based matrix, providing protection against environmental and gastrointestinal stresses and facilitating site-specific release within the GIT [39].

**Fig. 4:**
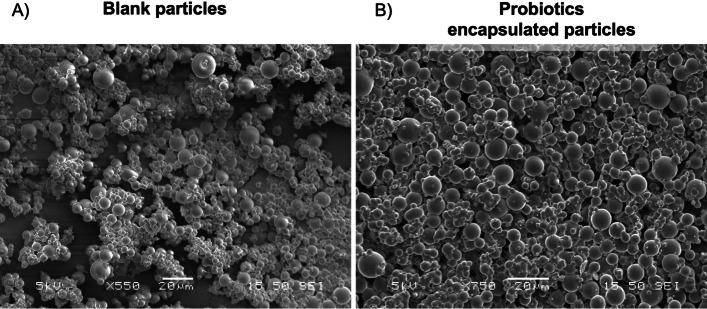
SEM images of (**A**) alginate control spray-dried microparticles and (**B**) alginate-encapsulated probiotics microparticles. Scale bar = 20 µm; magnification = 550 × for (**A**) and 750 × for (**B**)

**Fig. 5:**
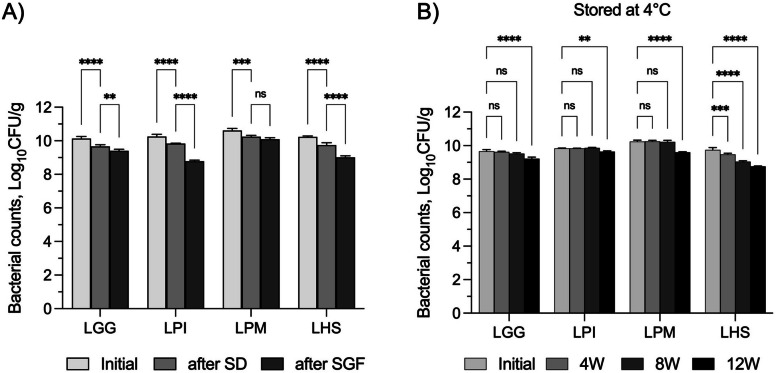
Survivability of the spray dried alginate encapsulated probiotic strains: (**A)** following spray drying and 2-h exposure to SGF, and (**B**) after 12 weeks of encapsulation and storage at 4 °C. *P < 0.05, **P < 0.01, ***P < 0.001, ****P < 0.0001 using one-way ANOVA and post-hoc Tukey test

Figure 4 presents electron micrographs of the spray-dried alginate particles, both control alginate and the alginate encapsulated probiotics. All microparticles were uniformly spherical and measured ≤ 10 µm in size. This small particle size is ideal for ensuring uniform mixing with fish feed ingredients without compromising the feed's palatability. Next, the survivability of the encapsulated probiotics was tested both after the spray-drying process and upon exposure to gastric conditions (pH ~ 2). The results of these survivability experiments are depicted in Fig. 5A. Some probiotics experienced a reduction of at least 0.5 log_10_ in CFU, with *L. plantarum* sp. showing comparatively less reduction in CFU. This loss is within the expected range during the encapsulation process. Additionally, the probiotics were exposed to the SGF (pH ~ 2) to simulate the environment of the GIT. *L. plantarum* sp. exhibited the highest tolerance to the SGF compared to the other strains. No significant difference was observed in log_10_ CFU counts after spray drying (SD) and after exposure to the SGF, while the other probiotic strains showed a significant reduction in CFU.

The encapsulated strains were also subjected to a 12-week storage stability test to assess the shelf life of the probiotics. As shown in Fig. 5B, all strains demonstrated good survivability when stored at 4 °C for up to eight weeks, although some loss was observed after 12 weeks. The survivability of probiotics under acid exposure was also evaluated after storage. *L. plantarum* sp. had the highest survival after SGF exposure post-storage compared to all other strains. *L. plantarum* sp. was selected for subsequent feeding trials.

### Growth performance trials

#### Pelletizing functional feeds

To investigate the combinatorial effects of probiotics and curcumin on the growth performance of Asian seabass fingerlings, a feeding trial was designed using five distinct functional feed groups: EP, FC, FPFC, EPFC, and a control (non-supplemented) group. The unencapsulated probiotics (FP) were produced through spray drying of the probiotic solution to have an effective comparison against the encapsulated probiotic (EP) samples. Spray drying also enables large-scale production of these probiotics for commercial use. The survivability of the spray-dried probiotics was assessed across multiple batches, yielding an average survivability of approximately 10^10^ CFU/g. These probiotic powders were then incorporated into the respective feed formulations and pelletized to the appropriate size. The final concentration of viable probiotics in the diets was approximately ~ 6.7 × 10^9^ CFU/kg of feed.

All diets were analysed for their composition, and the resulting nutritional profiles of the functional feeds were found to be comparable (Additional file 1: Table S2), ensuring that any observed differences in fish performance could be attributed to the functional additives rather than to nutritional imbalances.

#### Growth performance with functional feeds

**Fig. 6:**
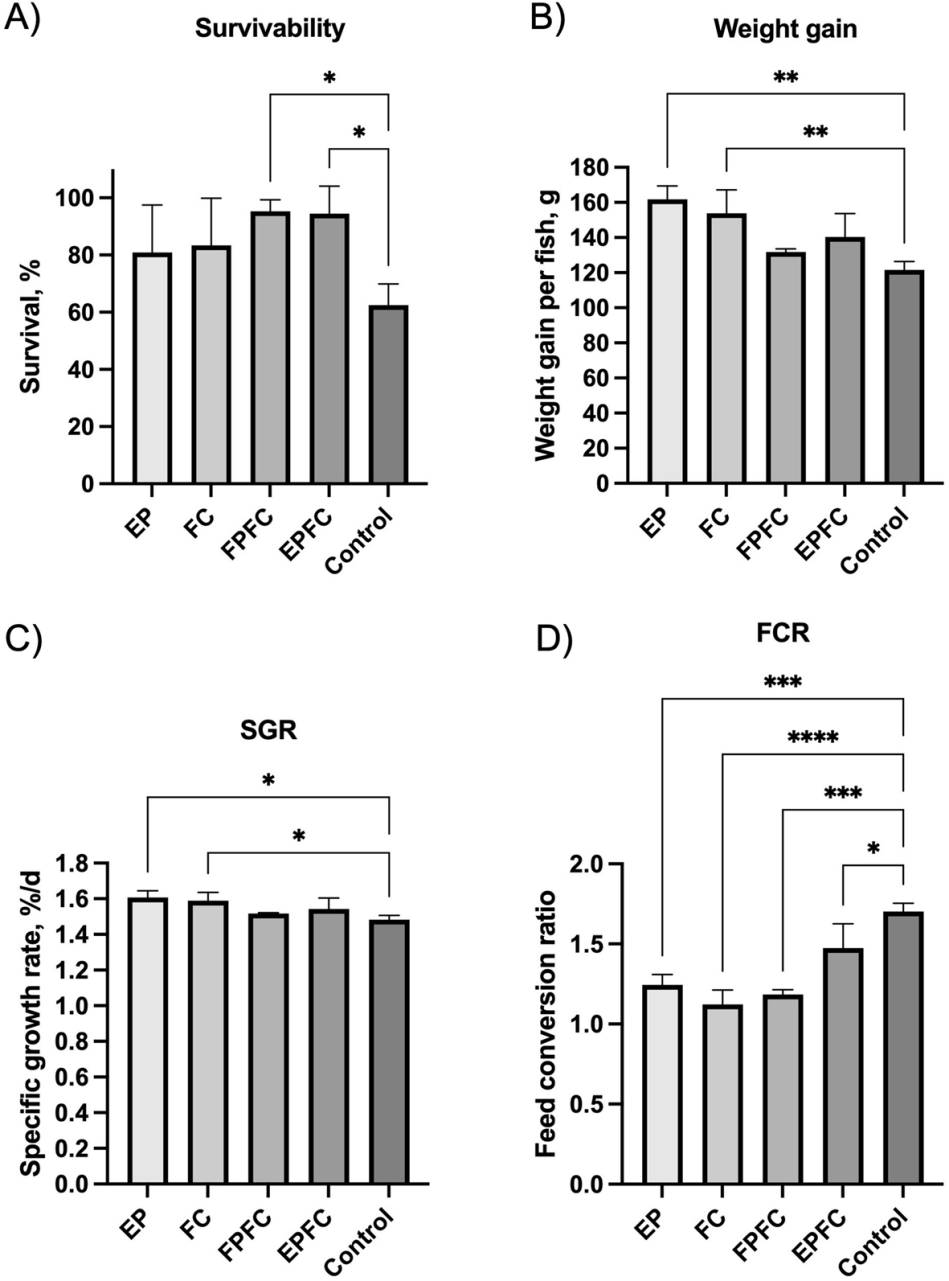
Effects of the functional feeds on the growth performance of Asian seabass fingerlings from week 0 to week 28. **A** Survivability. **B** Weight gain of fish. **C** Specific growth rate (SGR). **D** Feed conversion ratio (FCR). ^*^P < 0.05, ^**^P < 0.01, ^***^P < 0.001, ^****^P < 0.0001 using one-way ANOVA and post-hoc Tukey test

To evaluate the effects of functional feed supplementation on the growth performance of Asian seabass fingerlings, fish were fed with the formulated diets throughout the feeding trial. Survival analysis indicated that the control group, which received no functional additives, exhibited the lowest survivability among all groups (Fig. 6A; Table 4). In contrast, all groups receiving functional feeds showed improved survival rates, with a significant difference demonstrated for both combinations of probiotic with curcumin.

**Table 4:** Effects of the functional feeds on the growth performance of Asian seabass fingerlings from week 0 to week 28

Groups	EP	FC	FPFC	EPFC	Control	*P*-values
Survivability, %	80.9 ± 16.6^a^	83.3 ± 16.5^a^	95.2 ± 4.1^b^	94.4 ± 9.6^b^	62.5 ± 7.4^a^	0.0417
ABWG, g	161.7 ± 7.7^b^	153.8 ± 13.3^b^	131.8 ± 1.8^a^	140.4 ± 13.3^a^	121.6 ± 4.7^a^	0.0024
SGR, %/d	1.60 ± 0.04^b^	1.59 ± 0.05^b^	1.52 ± 0.0^a^	1.54 ± 0.06^a^	1.49 ± 0.02^a^	0.0181
FCR	1.25 ± 0.06^b^	1.12 ± 0.09^b^	1.19 ± 0.03^b^	1.47 ± 0.15^b^	1.71 ± 0.05^a^	< 0.0001

*ABWG* Average body weight gain, *SGR* Specific growth rate, *FCR* Feed conversion ratio. Values are presented as mean ± SD (*n* = 3). Values in the same row superscripted with different lowercase letters indicate significant differences (*P* < 0.05). *P*-values were calculated using one-way ANOVA followed by Tukey’s HSD post hoc test

Among the functional groups, fish fed with EP exhibited the most pronounced growth response, achieving a significant body weight gain of 33.0% ± 6.3% compared to the control group (Fig. 6B). The group supplemented with free curcumin (FC) demonstrated the next highest gain, with a 26.5% ± 10.9% increase. The combination diet groups FPFC and EPFC also promoted growth, resulting in body weight gains of approximately 8.4% ± 1.5% and 15.4% ± 11.0%, respectively. Additionally, all functional feed groups outperformed the control in terms of FCR and SGR, indicating improved feed efficiency and overall growth dynamics (Fig. 6C, D and Table 4).

### Pathogen challenge test

#### Challenge test against *V. parahaemolyticus*

**Fig. 7:**
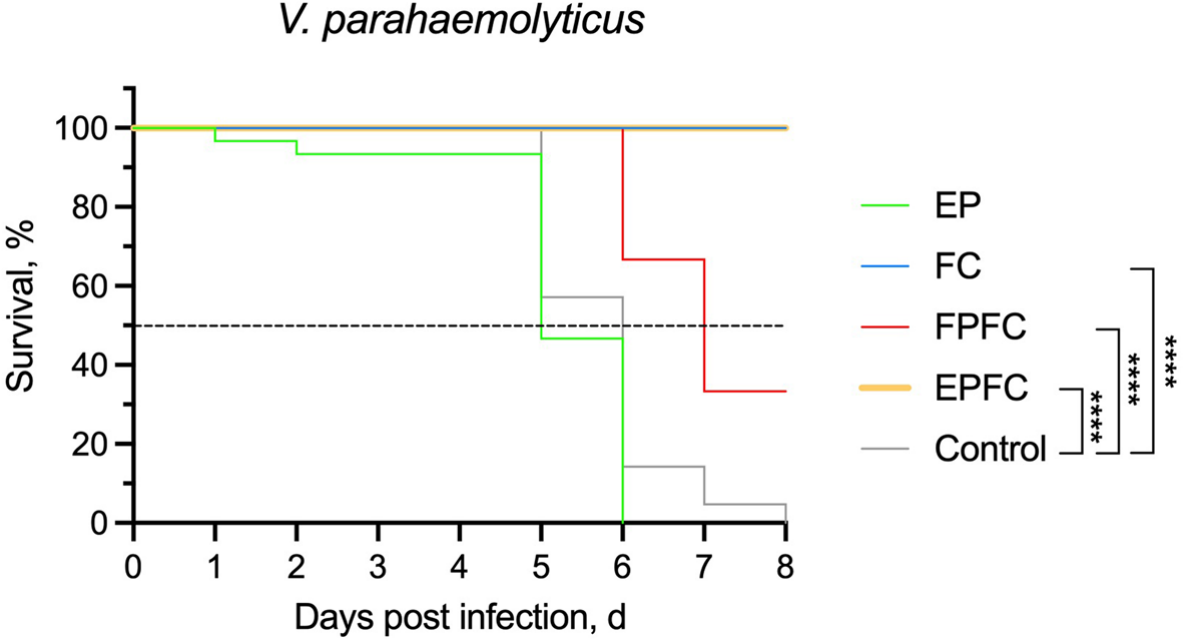
Kaplan–Meier survival curves of Asian sea bass from the five experimental groups following challenge with *V. parahaemolyticus*. Statistical significance was determined using the Log-rank (Mantel–Cox) test (^*^P < 0.05, ^**^P < 0.01, ^***^P < 0.001, ^****^P < 0.0001)

Following the 28-week feeding trial with the respective functional diets, a pathogen challenge test was conducted to evaluate the protective efficacy of the treatments against *Vibrio* infection. Fish from each dietary group were exposed to a lethal dose 50% (LD₅₀) concentration of *V. parahaemolyticus*, approximately 10^8^ CFU/mL, administered via i.p. injection. Figure 7 showed that the control group exhibited the expected LD_50_ response, with 50% mortality occurring within the observation period. Among the treatment groups, fingerlings fed with the EPFC and FC diets demonstrated complete protection, achieving 100% survival. The FPFC group showed partial protection, with a survival rate of 33%. In contrast, both the control and EP groups exhibited the lowest survival (Fig. 7). In summary, the survival rates of the experimental groups, in descending order, are as follows:

$$EPFC = FC (100\% survival)> FPFC (33\% survival)> Control = EP$$

These findings demonstrate the potential of curcumin, particularly when used alone or in combination with encapsulated probiotics, to enhance disease resistance in Asian seabass fingerlings.

#### Challenge test against *S. iniae*

Pathogen challenge was also conducted to evaluate the efficacy of the functional feeds in protecting Asian seabass fingerlings against *S. iniae* infection. The challenge protocol was performed similarly to the *Vibrio* challenge test but incorporated additional steps to enhance infection efficiency due to the lower virulence observed in the preliminary LD_50_ trial with *S. iniae*.

In the LD_50_ trial, i.p. injection of 10^7^ CFU/fish *S. iniae* resulted in only 42% cumulative mortality, suggesting suboptimal infection. To address this, a hybrid challenge model was employed in which fish were sedated, subjected to superficial skin abrasion, and then injected i.p. with approximately 2.4 × 10^7^ CFU/fish. This method improved the consistency and severity of infection across experimental groups.

**Fig. 8:**
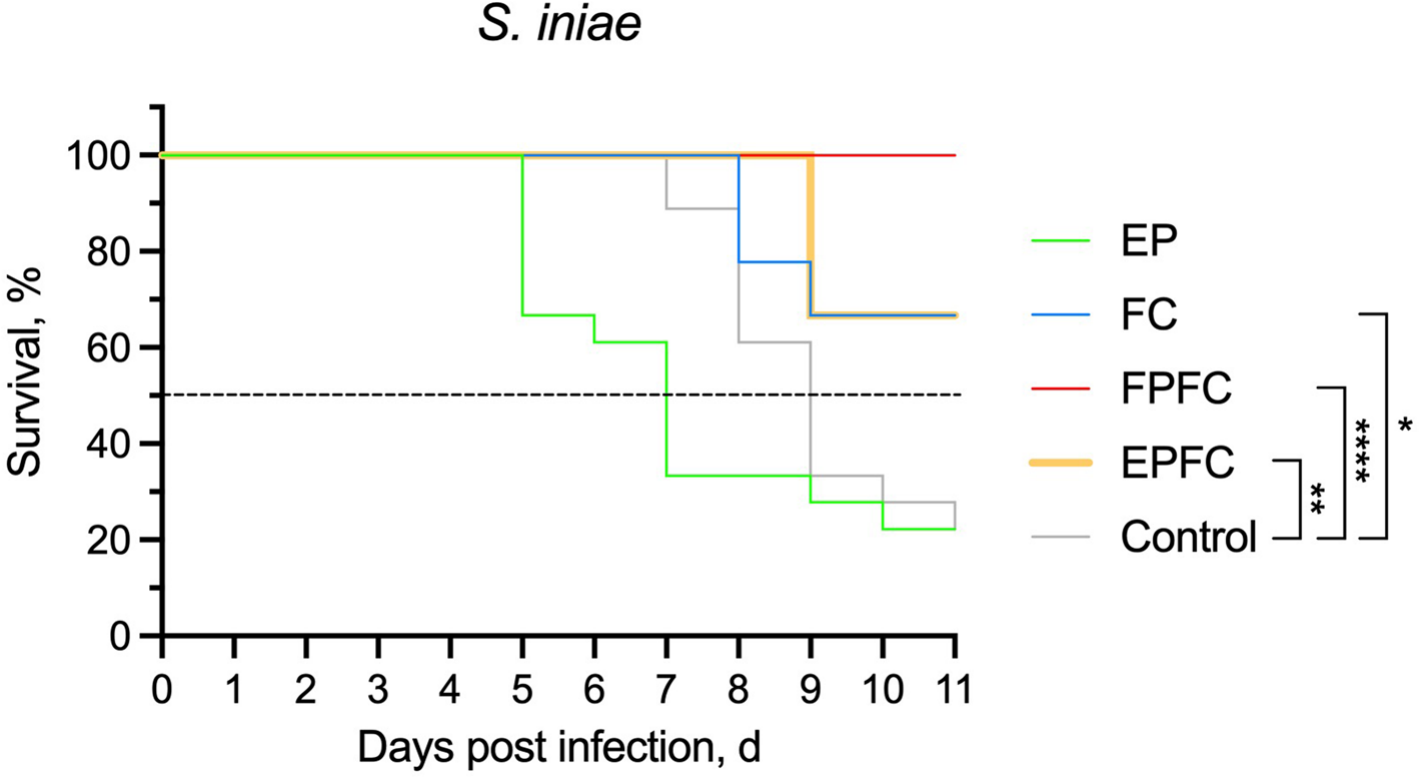
Kaplan–Meier survival curves of Asian sea bass from the five experimental groups following challenge with *S. iniae*. Statistical significance was determined using the Log-rank (Mantel–Cox) test (^*^P < 0.05, ^**^P < 0.01, ^***^P < 0.001, ^****^P < 0.0001)

The results showed that the FPFC group conferred the highest level of protection against *S. iniae*, achieving a 100% survival rate (Fig. 8). This was followed by the EPFC and FC groups, each demonstrating a survival rate of 66.7%. In contrast, the EP and control groups exhibited the highest mortality, with survival rates significantly lower than those of the curcumin-supplemented treatments.

In summary, the survival rates of the experimental groups, in descending order, are as follows:

$$FPFC (100\% survival)> EPFC = FC (66.7\% survival)> Control = EP$$

These findings highlight the promising role of curcumin-containing functional feeds—particularly when combined with probiotics—in enhancing the resistance of Asian seabass fingerlings to *S. iniae* infection.

## Discussion

This study evaluated the potential of probiotics and curcumin to enhance growth and disease resistance in Asian seabass fingerlings. The findings provide new insights into how functional feed additives can be strategically combined to improve health and productivity in aquaculture systems.

Several probiotic strains demonstrated strong inhibitory activity against *S. iniae* and *V. parahaemolyticus*. The inhibition was not related to pH, suggesting that antimicrobial metabolites such as bacteriocins, hydrogen peroxide, or organic acids were involved. These observations are consistent with previous reports that lactic acid bacteria (LAB) suppress aquaculture pathogens through multiple mechanisms, including competition for nutrients, production of antimicrobial compounds, and direct growth inhibition [53, 54]. Together, these results reinforce the role of probiotics as natural biocontrol agents in aquaculture.

Curcumin was also assessed for its antimicrobial activity. It selectively inhibited both pathogens while maintaining the viability of probiotic strains. This is consistent with previous studies whereby curcumin suppresses harmful bacteria without adversely affecting beneficial microorganisms [52, 55]. The tolerance of *Lactobacillus *spp*.* to curcumin may be attributed to their natural association with turmeric rhizomes [56, 57]. At the tested concentrations of 0.75–1.5 mg/mL, curcumin proved effective against both pathogens, supporting its suitability as a co-supplementation agent. Importantly, curcumin’s antimicrobial action may involve disruption of bacterial membranes and interference with quorum sensing and biofilm formation [58, 59]. Taken together, these observations suggest that curcumin can complement probiotic activity, broadening the antimicrobial spectrum while maintaining beneficial microbes.

When probiotics and curcumin were combined, stronger antimicrobial effects were observed than with either treatment alone, highlighting their complementary activities. This synergy supports the concept that integrating bioactive compounds with probiotics can deliver comprehensive protection, a strategy increasingly emphasized in functional feed research.

Beyond direct antimicrobial effects, probiotic performance was also observed to be strongly influenced by the mode of delivery, with encapsulation showing clear advantages. Encapsulation improved probiotic stability, survivability under gastric stress, and storage stability [60]. Among the tested strains, *L. plantarum *spp*.* displayed the greatest resilience, consistent with reports of its robust stress tolerance [61, 62]. Furthermore, the small particle size of encapsulated probiotics facilitates uniform mixing with feed ingredients without affecting palatability [63]. These characteristics support encapsulation as a practical and effective approach for delivering viable probiotics to the gastrointestinal tract of aquaculture species.

The benefits of encapsulated probiotics and curcumin were also reflected in fish performance outcomes. Encapsulated probiotics enhanced growth, while curcumin improved pathogen resistance. The high survival and FCR observed in EPFC suggest that improved probiotic viability and targeted delivery, together with curcumin’s antioxidant, anti-inflammatory, and antimicrobial activities, stimulated immune activity in fingerlings. Increased immune function likely elevated metabolic demands, diverting energy from growth toward defense processes. Such trade-offs between immunity and growth have been reported previously in fish, where immune activation carries metabolic costs [64, 65]. This observation highlights that functional feed outcomes depend on how energy is allocated between growth and immunity under different physiological conditions. When immune responses are strongly activated, survival benefits may be prioritized over growth efficiency, whereas in situations where immune demands are lower, functional feeds more consistently support improvements in growth and feed conversion efficiency. Evidence from previous studies supports this interpretation. Diets containing probiotics such as *Bacillus* strains have improved growth and feed efficiency in aquaculture species when immune demands were not excessive [66].

The enhanced growth and survival associated with diets containing encapsulated probiotics, either alone or in combination with curcumin, likely arise from coordinated effects on gut microbiota composition, digestive function, and immune competence. Probiotics are known to modulate intestinal microbial communities, enhance resistance to pathogen colonization, and promote digestive enzyme activities, thereby supporting improved nutrient utilization and feed efficiency [63, 67]. In parallel, probiotics stimulate key innate immune parameters, including lysozyme and complement activity, which can reduce pathogen burden and lower the energetic costs of sustained immune activation [68]. Encapsulation improved probiotic survival through the gastrointestinal tract, amplifying these effects relative to non-encapsulated formulations.

Curcumin exerts its effects primarily via antioxidant and immunomodulatory pathways. Previous studies have demonstrated that dietary curcumin elevates antioxidant enzyme activities, including superoxide dismutase, catalase, and glutathione peroxidase, while reducing lipid peroxidation markers such as malondialdehyde, thereby mitigating oxidative stress and preserving cellular integrity in finfish [31, 35, 47]. Curcumin has also been reported to improve digestive enzyme activities and intestinal morphology, enhancing nutrient digestion and absorption efficiency [47, 69]. By reducing oxidative and inflammatory burdens, curcumin may lower maintenance energy requirements, allowing greater energy allocation toward growth and physiological stability [70]. The superior performance observed with the combined diets therefore likely reflects complementary actions of probiotics and curcumin on gut function, immunity, and metabolic balance.

Challenge trials further confirmed the protective value of curcumin-containing diets, although the extent and nature of protection differed between pathogens. Because functional feeds were withdrawn prior to pathogen challenge, and free probiotics in non-encapsulated formulations were likely inactivated by gastric conditions, the observed protective effects are best explained by longer-lasting physiological or immunological conditioning rather than acute antimicrobial activity.

Distinct protection patterns were evident between the two challenge models. Diets containing free probiotics combined with curcumin (FPFC) conferred stronger protection against *S. iniae*, a pathogen known to cause systemic infection following invasion of host tissues [71, 72]. This enhanced resistance may reflect immune priming by non-viable probiotic components, which are capable of activating pattern-recognition receptors and promoting systemic immune readiness [73, 74]. Curcumin may have further reinforced these responses through its anti-inflammatory and antioxidant effects, enabling a more effective host defense during systemic infection [75, 76].

In contrast, resistance to *V. parahaemolyticus*, a Gram-negative enteric pathogen that primarily targets mucosal surfaces, appeared to be more strongly associated with diets containing encapsulated probiotics and curcumin (EPFC). Encapsulation facilitated the delivery of viable probiotics to the intestine [77], where they could modulate microbiota composition, enhance mucin secretion, and improve epithelial barrier integrity [78]. Pre-challenge exposure to curcumin may also have induced lasting improvements in intestinal antioxidant capacity, inflammatory tone, and tight junction stability [75, 79], collectively strengthening mucosal defenses. These mechanistic differences, aligned with the distinct infection routes and pathogenic strategies of the two bacteria, could provide a plausible explanation for the contrasting protection patterns observed.

An unexpected observation was the increased mortality observed in fish receiving probiotics alone during the early post-challenge period. Similar findings have been reported in seabass supplemented with *Bacillus* species, where probiotic feeding increased mortality after *V. anguillarum* infection [66]. Although the mechanisms remain unclear, these findings suggest that probiotic benefits are context-dependent and shaped by factors including strain, host species, pathogen type, and dosage [80–82]. Importantly, co-supplementation with curcumin mitigated this negative outcome, indicating that combined functional strategies may provide greater stability and consistency in protective outcomes.

Overall, the results demonstrate that diets combining probiotics and curcumin (EPFC and FPFC) provide complementary benefits in aquaculture feeds. These combinations support growth under normal rearing conditions while enhancing immune competence and disease resistance during pathogen exposure. Taken together, these findings highlight the potential of curcumin-probiotic functional feeds as practical strategies for improving fish health and productivity in sustainable aquaculture systems.

## Conclusions

This study demonstrated that functional feeds combining probiotics and curcumin can enhance the growth, immunity, and pathogen resistance of Asian seabass fingerlings, particularly against *S. iniae* and *V. parahaemolyticus*. Encapsulation improved probiotic stability and delivery, supporting growth performance, while curcumin supplementation strengthened disease resistance. Notably, co-supplementation with probiotics and curcumin (EPFC and FPFC) conferred synergistic protection against pathogen challenges, highlighting their value in disease-prone aquaculture settings. Overall, these findings demonstrate the potential of curcumin-probiotic functional feeds as a sustainable alternative to antibiotics, providing a scalable strategy to improve fish health, productivity, and resilience while supporting responsible aquaculture practices. As these results were obtained under controlled laboratory conditions, validation in large-scale aquaculture trials is essential. Future research should define strain-specific effects, refine dosing and feed strategies, evaluate economic feasibility, and elucidate underlying mechanisms, particularly interactions with host immunity and the gut microbiome, to advance practical application in commercial aquaculture.

## Supplementary Information

Additional file 1: Table S1. Growth conditions of probiotic strains tested. Table S2. Compositional analysis of the feeds used for the feeding trial.

## Data Availability

The datasets presented in this study are included in this article and are available from the corresponding author upon reasonable request.

## Abbreviations

ABSL-2: Animal biosafety level 2
ATCC: American type culture collection
CFU: Colony forming units
DMSO: Dimethyl sulfoxide
EP: Encapsulated probiotic
EPFC: Combination of encapsulated probiotics and free curcumin
FC: Free curcumin
FCR: Feed conversion ratio
FBW: Final body weight
FI: Estimated feed intake
FPFC: Combination of free probiotics and free curcumin
GIT: Gastrointestinal tract
HSD: Honestly significant difference (Tukey’s HSD post hoc test)
IACUC: Institutional Animal Care and Use Committee
IBW: Initial body weight
i.p.: Intraperitoneal
LAB: Lactic acid bacteria
LAM: *Limosilactobacillus amylovorus*
LAS: *Lactobacillus acidophilus*
LFM: *Limosilactobacillus fermentum*
LGG: *Lactobacillus rhamnosus* GG
LHI: *Lentilactobacillus hilgardii*
LHS: *Lactobacillus helveticus*
LKI: *Lentilactobacillus kefiri*
LPI: *Lacticaseibacillus paracasei*
LPM: *Lactiplantibacillus plantarum*
LRI: *Limosilactobacillus reuteri*
LOS: *Limosilactobacillus oris*
LSS: *Liquorilactobacillus satsumensis*
LD50: 50% Lethal dose
MRS: De Man, Rogosa and Sharpe
MIC: Minimum inhibitory concentration
NB: Nutrient Broth
NSS: Normal saline solution
PBS: Phosphate buffered saline
RAS: Recirculating aquaculture systems
SCELSE: Singapore Centre for Environmental Life Sciences Engineering
SD: Standard deviation
SGF: Simulated gastric fluid
SGR: Specific growth rate
SIF: Simulated intestinal fluid
WG: Weight gain
WC: Wilkins Chalgren Anaerobe
