# Supplementary material for: Combinatorial supplementation of fish feeds enhanced growth performance and disease resilience in aquaculture
Source: J Anim Sci Biotechnol. 2026 Mar 8;17:41. doi: 10.1186/s40104-026-01357-3 (PMC12967729; doi:10.1186/s40104-026-01357-3)
Supplement: Supplementary file 1 — Additional file 1: Table S1. Growth conditions of probiotic strains tested. Table S2. Compositional analysis of the feeds used for the feeding trial. [file 40104_2026_1357_MOESM1_ESM.docx]

**Supplementary Materials**

**Combinatorial supplementation of fish feeds enhanced growth performance and disease resilience in aquaculture**

**The-Thien Tran^1^, Manish Mahotra^1^, Kaarunya Sampathkumar^1^, Wenrui Li^1^, Hong Yu^1^, Ling Xin Yong^1^, Li Ling Tan^1^, Mingyue Sun^1^, Patricia Lynne Conway^2,3^, Say Chye Joachim Loo^1,2,4*^**

^1^School of Materials Science and Engineering, Nanyang Technological University, 50 Nanyang Avenue, 639798 Singapore

^2^Singapore Centre for Environmental Life Sciences Engineering (SCELSE), Nanyang Technological University, 60 Nanyang Drive, 637551 Singapore

^3^School of Biological, Earth and Environmental Sciences, The University of New South Wales, Sydney, NSW 2052 Australia

^4^Lee Kong Chian School of Medicine, Nanyang Technological University, 11 Mandalay Road, 308232 Singapore

Additional file 1: Table S1. Growth conditions of probiotic strains tested.

| **No.** | **Probiotic species** | **Growth conditions** | | | |
| --- | --- | --- | --- | --- | --- |
|  |  | **Media** | **Temperature**  **(°C)** | **Aerobic/ anaerobic (OX/ANA)** | **Incubation duration (days)** |
| 1 | *Lactobacillus acidophilus* sp. | MRS | 37 | ANA | 1 |
| 2 | *Lacticaseibacillus paracasei* spp. | MRS | 37 | ANA | 1 |
| 3 | *Limosilactobacillus reuteri* sp. | MRS | 37 | ANA | 2 |
| 4 | *Lactobacillus amylovorus* sp. | MRS | 37 | ANA | 2 |
| 5 | *Limosilactobacillus oris* sp. | MRS | 37 | ANA | 2 |
| 6 | *Lactiplantibacillus plantarum* sp. | MRS | 37 | OX | 1 |
| 7 | *Limosilactobacillus fermentum* spp. | MRS | 37 | ANA | 1 |
| 8 | *Liquorilactobacillus satsumensis* spp. | MRS | 30 | ANA | 3 |
| 9 | *Lentilactobacillus kefiri* sp. | MRS | 30 | ANA | 3 |
| 10 | *Lactobacillus helveticus* sp. | MRS | 30 | ANA | 3 |
| 11 | *Lentilactobacillus hilgardii* sp. | MRS | 30 | ANA | 3 |
| 12 | *Lacticaseibacillus rhamnosus* GG | MRS | 37 | OX | 1 |

MRS = de Man, Rogosa and Sharpe medium; OX = aerobic conditions; ANA = anaerobic conditions. Incubation duration indicates the number of days required to observe visible growth under the specified conditions.

Additional file 1: Table S2. Compositional analysis of the feeds used for the feeding trial.

|  | EP (%) | FC (%) | FPFC (%) | EPFC (%) | Control (%) |
| --- | --- | --- | --- | --- | --- |
| Protein | 48.70 | 48.80 | 48.20 | 48.10 | 49.40 |
| Fat | 12.50 | 11.80 | 10.80 | 10.20 | 10.50 |
| Fibre | 0.33 | 0.31 | 0.32 | 0.25 | 0.31 |
| Ash | 5.09 | 5.13 | 5.09 | 5.09 | 5.21 |
| Moisture | 8.42 | 9.04 | 9.22 | 9.65 | 7.99 |
| Carbohydrates | 24.96 | 24.92 | 26.37 | 26.71 | 26.59 |

Values are expressed as percentages on a dry matter basis. FP = free probiotic; EP = encapsulated probiotic; FC = free curcumin; FPFC = free probiotic and free curcumin; EPFC = encapsulated probiotic and free curcumin. Control refers to the basal diet without functional additives.
